# Former Abusers of Anabolic Androgenic Steroids Exhibit Decreased Testosterone Levels and Hypogonadal Symptoms Years after Cessation: A Case-Control Study

**DOI:** 10.1371/journal.pone.0161208

**Published:** 2016-08-17

**Authors:** Jon Jarløv Rasmussen, Christian Selmer, Peter Busch Østergren, Karen Boje Pedersen, Morten Schou, Finn Gustafsson, Jens Faber, Anders Juul, Caroline Kistorp

**Affiliations:** 1 Department of Internal Medicine, Copenhagen University Hospital, Herlev, Denmark; 2 Department of Urology, Copenhagen University Hospital, Herlev, Denmark; 3 Department of Internal Medicine, Copenhagen University Hospital, Slagelse, Denmark; 4 Department of Cardiology, Copenhagen University Hospitals, Herlev and Gentofte, Denmark; 5 Department of Cardiology, the Heart Centre, Rigshospitalet, Copenhagen, Denmark; 6 Department of Growth and Reproduction, Rigshospitalet, University of Copenhagen, Denmark; University of Sydney, AUSTRALIA

## Abstract

**Aims:**

Abuse of anabolic androgenic steroids (AAS) is highly prevalent among male recreational athletes. The objective of this study was to investigate the impact of AAS abuse on reproductive hormone levels and symptoms suggestive of hypogonadism in current and former AAS abusers.

**Methods:**

This study had a cross-sectional case-control design and involved 37 current AAS abusers, 33 former AAS abusers (mean (95%CI) elapsed duration since AAS cessation: 2.5 (1.7; 3.7) years) and 30 healthy control participants. All participants were aged 18–50 years and were involved in recreational strength training. Reproductive hormones (FSH, LH, testosterone, inhibin B and anti-Müllerian hormone (AMH)) were measured using morning blood samples. Symptoms of hypogonadism (depressive symptoms, fatigue, decreased libido and erectile dysfunction) were recorded systematically.

**Results:**

Former AAS abusers exhibited significantly lower median (25^th^ –75^th^ percentiles) total and free testosterone levels than control participants (total testosterone: 14.4 (11.9–17.7) nmol/l vs. 18.8 (16.6–22.0) nmol/l) (P < 0.01). Overall, 27.2% (13.3; 45.5) of former AAS abusers exhibited plasma total testosterone levels below the lower reference limit (12.1 nmol/l) whereas no control participants exhibited testosterone below this limit (P < 0.01). Gonadotropins were significantly suppressed, and inhibin B and AMH were significantly decreased in current AAS abusers compared with former AAS abusers and control participants (P < 0.01). The group of former AAS abusers had higher proportions of participants with depressive symptoms ((24.2%) (11.1; 42.2)), erectile dysfunction ((27.3%) (13.3; 45.6)) and decreased libido ((40.1%) (23.2; 57.0)) than the other two groups (trend analyses: P < 0.05).

**Conclusions:**

Former AAS abusers exhibited significantly lower plasma testosterone levels and higher frequencies of symptoms suggestive of hypogonadism than healthy control participants years after AAS cessation. Current AAS abusers exhibited severely decreased AMH and inhibin B indicative of impaired spermatogenesis.

## Introduction

Anabolic androgenic steroids (AAS) comprise testosterone and its synthetic derivatives. These compounds have been used for decades by professional athletes to enhance muscle strength and performance [1, 2]. The setting of AAS abuse has changed within recent years. A recent meta-analysis estimated the lifetime prevalence of AAS abuse worldwide is 6.4% among men and 18.4% among recreational athletes [3]. Moreover, a previous study suggested that prior AAS abuse was the most frequent cause of profound hypogonadism among young men (43%) [4]. These findings indicate AAS abuse is now prevalent in the broader population.

Ongoing AAS abuse causes dramatic increases in plasma androgen levels that ultimately facilitate severe hypothalamic-pituitary-gonadal (HPG)-axis suppression due to negative feedback mechanisms involving testosterone and its metabolites [5]. HPG-axis inhibition may cause long-lasting spermatogenesis inhibition and reductions in biomarkers of Sertoli-cell function, anti-Müllerian hormone (AMH) and inhibin B. However, information regarding the impact of AAS abuse on these reproductive hormones is very limited [6, 7].

Anabolic androgenic steroid-induced hypogonadism (ASIH) is common among former AAS abusers and usually presents as hypogonadotropic hypogonadism due to abrupt decreases in plasma androgen levels following AAS cessation [1, 2, 5, 8]. Scientific data on ASIH are limited, but the condition is characterised by symptoms and signs of hypogonadism such as: testicular atrophy, low plasma testosterone levels, impaired spermatogenesis, erectile dysfunction, fatigue, decreased libido and depressive symptoms; and is considered to resolve spontaneously within 6 to 12 months [2, 5]. Studies investigating the recovery phases of young men with ASIH are, to our knowledge, virtually non-existent. A growing number of studies reporting cases, in which ASIH manifestations persisted years after AAS cessation, suggest ASIH is a more permanent condition in a substantial proportion of former AAS abusers [9–16]. This emerging group of young men may become a considerable public health concern in the coming years.

The objective of this study was to compare the reproductive hormone levels and symptoms suggestive of hypogonadism in young men with histories of current and former AAS abuse with those of healthy age-matched men.

## Participants and Methods

### Study Design and Participants

We conducted a community-based cross-sectional case-control study in the greater Copenhagen area from November 2014 to December 2015. Young men (18–50 years) involved in recreational strength training were enrolled in one of the following three groups: 1) current AAS abusers 2) former AAS abusers who had discontinued AAS abuse ≥ 3 months before enrolling in the study and 3) age-matched healthy control participants who denied ever having used AAS. We did not apply specific inclusion criteria regarding weekly hours of recreational strength training, nor did we apply inclusion criteria pertaining to the extent of AAS abuse. Exclusion criteria for the three groups were: congenital hypogonadal conditions, medically prescribed testosterone therapy, known cardiovascular disease and diabetes mellitus. Participants were recruited primarily from fitness centres in the greater Copenhagen area and by internet advertising. The fitness centres included weightlifting gymnasiums which are not under surveillance by the Danish Antidoping Agency and are known to be frequented by AAS abusers. The advertisement was as follows (in Danish): ‘We are seeking young men for a research project; inclusion criteria: age 18–50 years and involved in recreational strength training or are currently using AAS or have previously used AAS’. The advertisement did not disclose the study entailed assessments of: androgen levels, fertility biomarkers, libido, erectile function or symptoms of depression or fatigue.

### Ethics

The study was performed in accordance with the Declaration of Helsinki and all relevant legal regulations in Denmark. Permission to conduct the study was granted by the Danish Data Protection Agency (HEH-2014-095, I-Suite: 03250) and ethical approval was granted by the Capital Regional Committee on Health Research Ethics in Denmark (H-3-2014-127). Oral and written informed consent was obtained from all participants prior to inclusion.

### Procedures

All procedures were performed during one visit at the Centre of Endocrinology and Metabolism, Department of Internal Medicine, Copenhagen University Hospital, Herlev, Denmark. Participants attended the research lab between 07:30 and 09:00 a.m. after a minimum of eight hours of overnight fasting. They were placed in the supine position for a minimum of 30 minutes. All blood samples were then collected via a cannula in the right median cubital vein.

One investigator (JJR) obtained a detailed AAS abuse history (total duration, compounds, doses, use of other performance enhancing drugs) during a clinical interview, using a structured questionnaire. We used total numbers of weeks of AAS abuse and total numbers of AAS compounds used as measures of the extent of AAS abuse. We did not calculate overall average AAS doses in the AAS participants because the pharmacodynamics and pharmacokinetics of AAS compounds can vary considerably depending on their chemical structures [1]. Furthermore, AAS abusers often use numerous AAS compounds and alter doses intermittently during a ‘cycle’ [17]. Other information such as medical history, illicit drug use, smoking habits, alcohol use, strength training history (total duration and weekly hours of training) and demographics were also obtained. Testicular size (ml) was assessed in all participant using Prader’s orchidometer, which has shown strong correlations with ultrasound testicular size estimations [18].

### Laboratory Analyses

Plasma total testosterone, androstendione, dehydroepiandrosteronsulfate (DHEAS) and 17-hydroxyprogesterone were all measured using liquid chromatography-mass spectrometry (LC-MS/MS), according to the CHS MSMS steroids kit (PerkinElmer, Massachusetts, USA). The inter-assay coefficient of variation (CV) was < 10.0% for all steroid hormones. Sexual hormone-binding globulin (SHBG) was analysed by sandwich chemiluminescence-based immunoassay (Siemens, Munich, Germany) and the CV was < 7.0%. Free testosterone was calculated using the method suggested by Bartsch [19]. Low reference limits (2.5 percentile) for plasma total testosterone in young men differ among studies depending on estimation in subgroups of nonobese eugonadal healthy young men (10.4–12.1 nmol/l) or more population-representative cohorts (6.6–7.4 nmol/l) [20–23]. Therefore, we assessed proportions of the control group and group of former AAS who exhibited low total testosterone levels using lower reference limits for both a subgroup of eugonadal nonobese healthy subgroup of young men (12.1 nmol) and a pooled population-representative cohort (6.6 nmol/l). Gonadotropins were measured by a sandwich electrochemiluminescence-immunoassay (ECLIA) (Cobas, Roche, Switzerland) and the CVs were < 7.0%. Serum inhibin B and anti-Müllerian hormone (AMH) levels were used as markers of Sertoli-cell function and spermatogenesis [24–26]. Serum was kept frozen at -80°C until needed for analysis on the same batch. Inhibin B was measured by a three-step sandwich-ELISA assay (inhibin B genII) (Beckman Coulter, California, USA). The intra-assay variations were < 10.3%, < 8.6% and < 7.6% at levels of 36.3, 111.4 and 616.4 pg/ml, respectively. Serum inhibin B is strongly associated with sperm concentrations and sperm counts especially up to a level of 150 pg/ml [25]. We used a reference limit of ≤ 92 pg/ml as cut-off for impaired spermatogenesis based on the results of a recent study [24]. AMH was analysed by a two–step sandwich electrochemiluminescence assay (Beckman Coulter, California, USA) and the intra-assay variations were < 1.77%, < 2.48% and < 2.39% at levels of 7.04, 34.88 and 105.66 pmol/l, respectively.

### Symptoms Indicating Hypogonadism

The Beck Depression Inventory-II (BDI-II) questionnaire was used to evaluate participants regarding the presence of depressive symptoms [27]. The BDI-II is a 21-question multiple-choice self-reported psychometric test, and each of its questions is scored using a scale ranging from 0 (minimum) to 3 (maximum). A total score ≤ 10 is considered normal, and the BDI-II is strongly correlated with other validated psychometric tests used in primary care settings [28]. Question number 21 of the BDI-II was used to assess libido among the participants and we considered a score < 1 normal. Erectile function was evaluated using the five-item version of the International Index of Erectile Function (IIEF-5) questionnaire [29]. The questionnaire is highly validated and consists of five questions scored using a scale ranging from 1 to 5. A total score ≥ 22 indicates normal erectile function. The Short Form-36 (SF-36) questionnaire was used to assess ‘energy/fatigue’. Lower scores were suggestive of more pronounced fatigue symptoms [30].

### Statistical Analyses

Categorical variables were compared using a chi-square test or Fisher’s exact test as appropriate. The non-parametric Cochran-Armitage trend test was used to assess trends in hypogonadal symptoms and impaired spermatogenesis across the groups. The ordinal ordering of the groups for trend analyses was specified a priori. Assumptions of normal distributions with respect to numerical variables were evaluated by histograms and by assessing the linearity of residuals in a quantile plot. Equality of variance was assessed using residuals of variables drawn against predicted values and using Levene’s test. Numerical variables were compared across the groups by analysis of variance (ANOVA) and presented as mean (standard error) if the assumptions of a normal distribution and equality of variance were fulfilled. Furthermore, numerical variables were compared pairwise among the three groups with Tukey’s post-hoc test. The non-parametric Kruskal-Wallis’ test (with Boneferroni’s post-hoc test) was used to compare non-normally distributed variables which could not be logarithmically transformed to an adequate normal distribution. These variables are presented as medians (25^th^– 75^th^ percentiles). We used piecewise linear regression (linear splines), allowing varying slopes, to model nonlinear associations. Missing data were rare (≈ 2% for questionnaires, none for all other data) and were addressed via multiple imputations using the fully conditional specification method [31]. P-values < 0.05 were considered statistically significant. All data were analysed using SAS version 9.4 (SAS Institute Inc., North Carolina, USA).

## Results

### Characteristics of Participants

A total of 37 current AAS abusers, 33 former AAS abusers and 31 control participants volunteered to participate in the study. One participant from the control group was excluded due to cryptorchidism which was diagnosed during the study, so 30 control participants were included in the final analyses. The participants in the three groups did not differ significantly with respect to age, smoking history, illicit drug abuse history, income or marital status, but current AAS abusers performed strength training more hours per week than participants in the other two groups (P < 0.05) (Table 1).

**Table 1:** Demographic characteristics and anabolic androgenic steroids (AAS) abuse in the three groups.

Variable	Control group	Current AAS abusers	Former AAS abusers	p-value
	n = 30	n = 37	n = 33	
*Demographic characteristics*				
Age (years) ¶	31.5 (1.2)	31.4 (1.4)	34.8 (1.2)	0.11
Recreational strength training (hours/week) ¶	6.5 (0.5)	9.2 (0.7) ***a***	6.9 (0.7)	0.01
Cohabiting (%)	73.3	67.6	57.6	0.41
Income (US $/year)	51,700 (5400)	43,000 (7700)	60,800 (7600)	0.35
University degree (%)	30.0 ***b***	0.0	0.0	< 0.01
History of smoking (%)	26.7	43.2	51.5	0.10
Alcohol intake < once /week (%)	56.7 ***c***	86.5	72.7	0.02
Experience with illicit drugs (%)	56.7	70.3	69.7	0.43
*Anabolic androgenic steroids abuse*				
Accumulated duration of AAS abuse (weeks)	-	142.3 (99.7–203.1)	111.8 (81.3–153.7)	0.32
AAS abuse during elapsed period (years)	-	5.7 (4.5–7.2)	6.3 (4.5–8.8)	0.46
Elapsed duration since AAS cessation (years)	-	-	2.5 (1.7–3.7)	-
Number of AAS compounds used (n) ●		8 (4–9)	6 (4–9)	0.32
*Post-cycle therapy*	-			
Regularly used hCG (%)	**-**	48.7	57.6	0.46
Regularly used aromatase inhibitors /antioestrogen (%)	**-**	48.7	33.3	0.19

Results are geometric means (95% confidence interval) unless otherwise stated.

¶ Mean (standard error)

● Median (25^th^– 75^th^ percentiles)

Tukey’s post-hoc test (mean and geometric mean) or Bonferroni’s post-hoc test (medians)

***a*** significant difference between current AAS abusers and the other two groups

***b*** significant difference between current AAS abusers and the other two groups

***c*** significant difference between control participants and current AAS abusers

A higher proportion of participants in the control group had a university degree than the participants in the other two groups (P < 0.01). The total duration of accumulated AAS abuse (geometric mean (95%CI)) noted among current AAS abusers (142.3 (99.7; 203.1) weeks) was not significantly different from that noted among former AAS abusers (111.8 (81.3; 153.7) weeks), and the numbers of AAS compounds used did also not differ between the two groups. The two groups reported previous and current experience with varying doses of numerous AAS compounds, of which testosterone esters, trenbolone, nandrolone, stanozolol, sustanon and boldenone were the most widely used (S1 Table). High proportions of both current and former AAS abusers reported regularly using hCG or aromatase inhibitors following AAS cycles. The elapsed duration since AAS cessation (geometric mean (95%CI)) was 2.5 (1.7; 3.7) years among former AAS abusers. None of these participants reported having used AAS within six months and only 15.2% (95%CI) (3.0; 27.4) reported elapsed time interval of 6–12 months since AAS cessation. Eleven former AAS abusers had previously been referred to an endocrine clinic for gynaecomastia, but none had been treated for gynaecomastia, hypogonadism or infertility. These participants did not differ from other former AAS abusers in terms of demographic characteristics, AAS abuse, laboratory results or frequency of hypogonadal symptoms.

### Reproductive Hormones

Testicular size differed significantly among the three groups. Current AAS abusers had the smallest testicular volume (12.2 (0.7) ml) and former AAS abusers had a volume of 17.4 (0.8) ml which was 4.8 (2.9; 6.8) ml smaller than that of the control participants who had largest testicular volume (Table 2). Plasma total and free testosterone levels were significantly lower among former AAS abusers than among control participants and current AAS abusers, the latter of whom exhibited significantly increased plasma testosterone levels, as expected. The 2.5^th^– 97.5^th^ percentiles for total testosterone ranged from 12.4–32.3 nmol/l among the control participants and 5.7–31.4 nmol/l among former AAS abusers.

**Table 2:** Reproductive hormone levels in the three groups.

Variable	Control group	Current AAS abusers	Former AAS abusers	p-value
	n = 30	n = 37	n = 33	
Testicular size (ml) ¶	22.3 (0.6) ***b***	12.2 (0.7)	17.4 (0.8)	< 0.01
P-total testosterone (nmol/l)	18.8 (16.6–22.0) ***b***	98.3 (47.4–122.7)	14.4 (11.9–17.7)	< 0.01
P-free testosterone (pmol/l)	480 (420–530) ***b***	3780 (1870–5500)	410 (320–480)	< 0.01
P-androstendione (nmol/l) ●	2.53 (2.27–2.82)	6.92 (5.41–8.84) ***a***	2.33 (2.06–2.63)	< 0.01
P-DHEAS (nmol/l) ¶	4805 (391)	4929 (490)	4348 (302)	0.55
P-SHBG (nmol/l) ●	33.3 (29.1–38.1)	8.4 (6.3–11.1) ***a***	26.2 (20.7–33.1)	< 0.01
P-17 hydroxyprogesterone (nmol/l) ●	2.88 (2.49–3.33)	0.14 (0.10–0.18) ***a***	2.42 (1.86–3.15)	< 0.01
P-FSH (U/l)	4.2 (3.2–5.7)	0.3 (0.1–0.4) ***a***	4.4 (3.3–6.2)	< 0.01
P-LH (U/l)	3.1 (2.5–3.9)	<0.1 (<0.1–0.1) ***a***	3.6 (2.2–4.3)	< 0.01
S-inhibin B (pg/ml) ¶	175 (9)	81 (8) ***a***	170 (11)	< 0.01
S-AMH (pmol/l) ●	49.5 (41.6–59.0)	21.6 (16.3–28.7) ***a***	44.7 (37.2–53.7)	< 0.01

Results are medians (25^th^– 75^th^ percentiles) unless otherwise stated.

¶ Mean (standard error)

● Geometric mean (95% confidence interval)

Tukey’s post-hoc test (mean and geometric mean) or Bonferroni’s post-hoc test (medians)

***a*** significant difference between the group of current AAS abusers and the two other groups

***b*** significant difference among all three groups

**AAS,** anabolic androgenic steroids, **AMH,** anti-Müllerian hormone; **DHEAS,** dehydroepiandrosteronsulfate; **FSH,** follicle-stimulating hormone; **LH,** luteinizing hormone; **P-**, plasma; **S-,** serum **SHBG,** sexual hormone-binding globulin.

A high percentage of participants in the group of former AAS abusers (27.2% (13.3; 45.5)) were below the lower reference limit for plasma total testosterone estimated in nonobese eugonadal healthy young men (12.1 nmol/l) whereas no participants in the control group (0.0% (0.0; 11.6)) were below this limit (P < 0.01). Further, among former AAS abusers 3.3% (0.01; 15.8) were below the lower reference limit for plasma total testosterone estimated in a pooled population-representative cohort (6.6 nmol/l). Plasma gonadotropins, SHBG, 17-hydroxyprogesterone, serum AMH and inhibin B did not differ significantly between former AAS abusers and control participants, but were markedly decreased among current AAS abusers (P < 0.01). There were higher percentages of participants with serum inhibin B levels below the limit of impaired spermatogenesis (92 pg/ml) among current AAS abusers (56.8% (39.5; 72.7)) and former AAS abusers (9.1% (1.9; 24.3)) than among control participants (3.3% (0.01; 17.2)) (trend analysis: P < 0.01).

**Fig 1:**
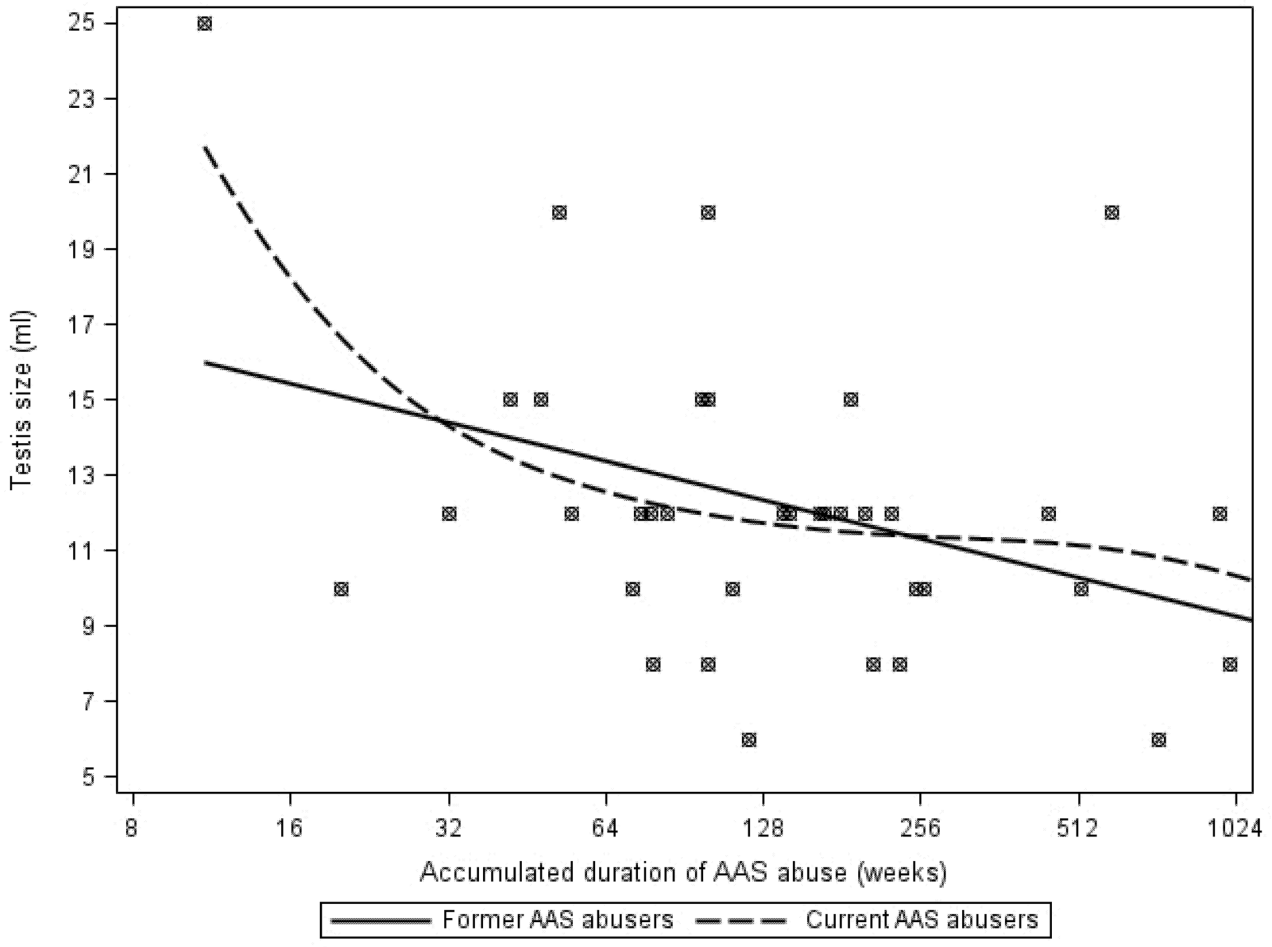
Association between accumulated duration of AAS abuse (log 2 scale) and testis size in current AAS abusers (spline function) and former AAS abusers. **Footnote: AAS,** anabolic androgenic steroids.

**Fig 2:**
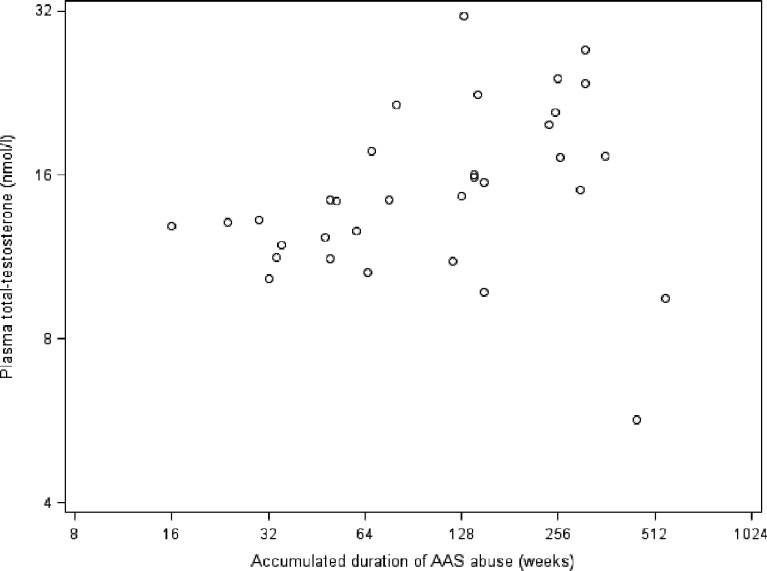
Association between accumulated duration of AAS abuse (log 2 scale) and plasma total testosterone levels (log 2 scale) in former AAS abusers. **Footnote: AAS,** anabolic androgenic steroids.

**Fig 3:**
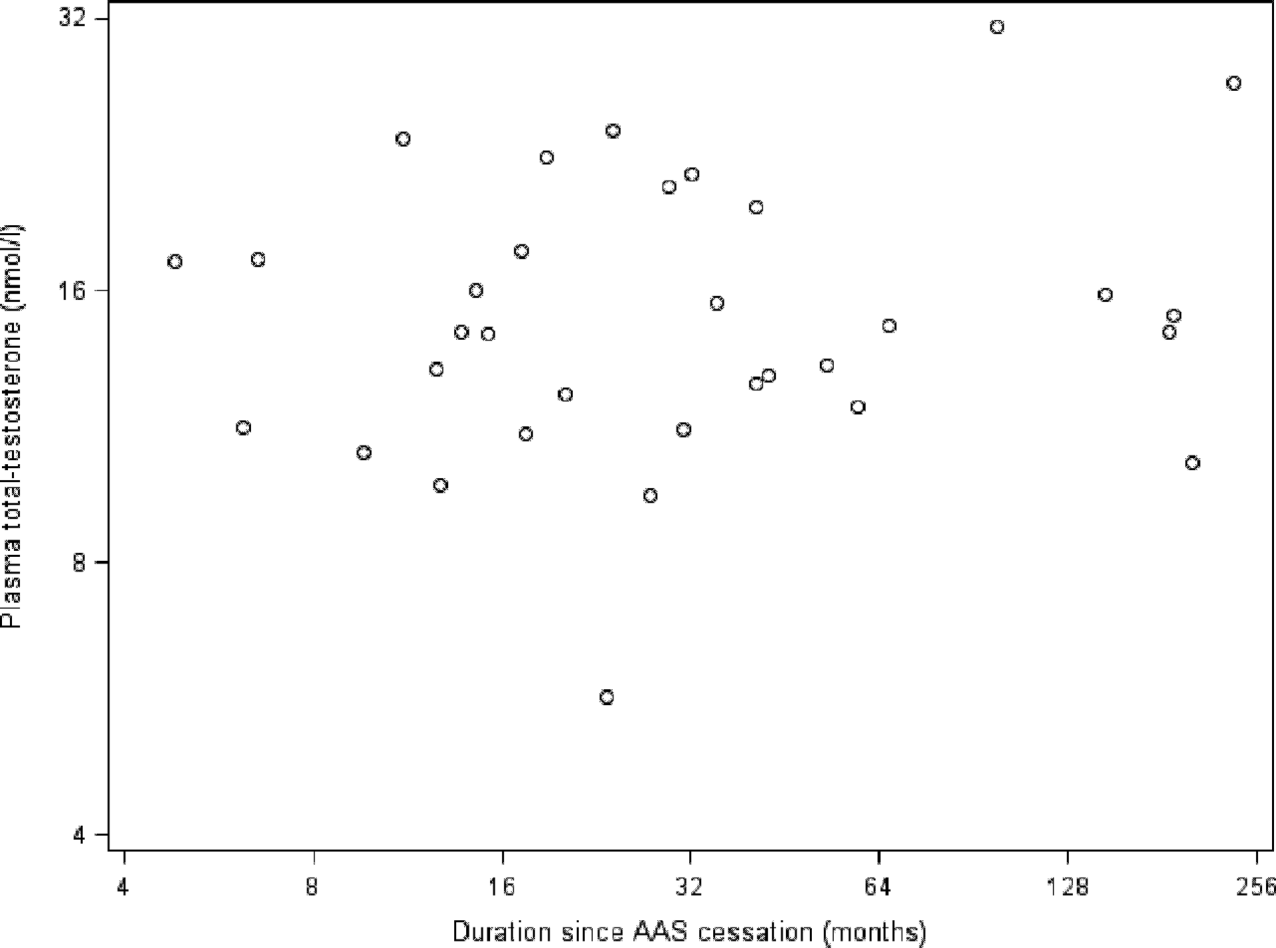
Association between elapsed duration since AAS cessation (log 2 scale) and plasma total testosterone levels (log 2 scale) in former AAS abusers. **Footnote: AAS,** anabolic androgenic steroids.

Accumulated duration of AAS abuse was associated with reduced testicular size in former abusers (log2 coefficient (B) (95%CI): -1.3 (-2.4; -0.2), P = 0.02) and current AAS abusers (during the initial 32 weeks of AAS abuse, spline function, log2 coefficient (B): -5.4 (-10.8; -0.02), P = 0.049) (Fig 1). We did not observe any significant associations between plasma total testosterone levels and accumulated duration of AAS abuse (log2 coefficient (B): 0.09 (-0.04; 0.22), P = 0.17) (Fig 2) or elapsed duration since AAS cessation (log2 coefficient (B): 0.05 (-0.7; 0.17), P = 0.42) (Fig 3) among former AAS abusers.

**Fig 4:**
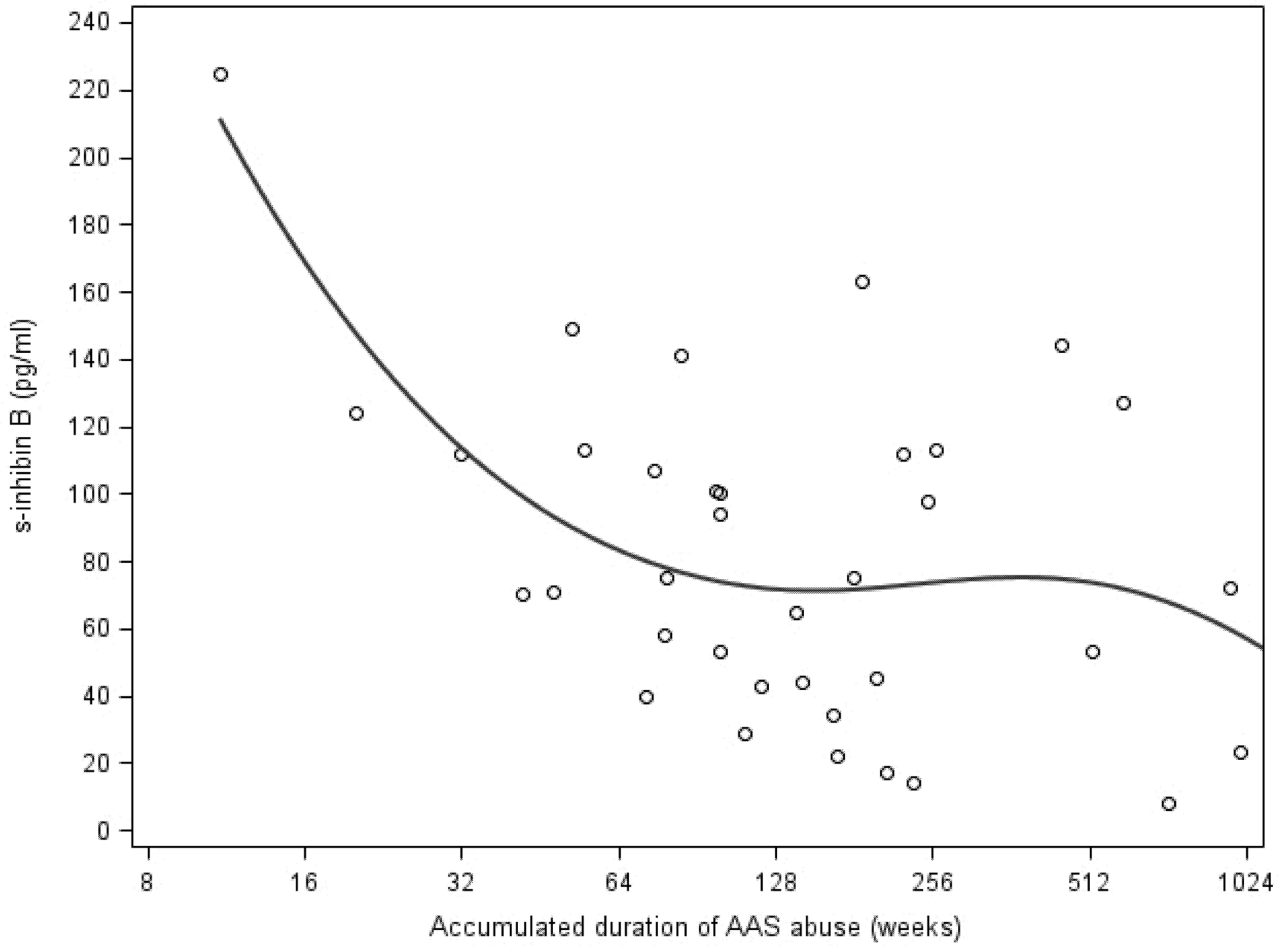
Association between accumulated duration of AAS abuse (log 2 scale spline function) and serum inhibin B levels in current AAS abusers. **Footnote: AAS,** anabolic androgenic steroids; **s-**, serum.

**Fig 5:**
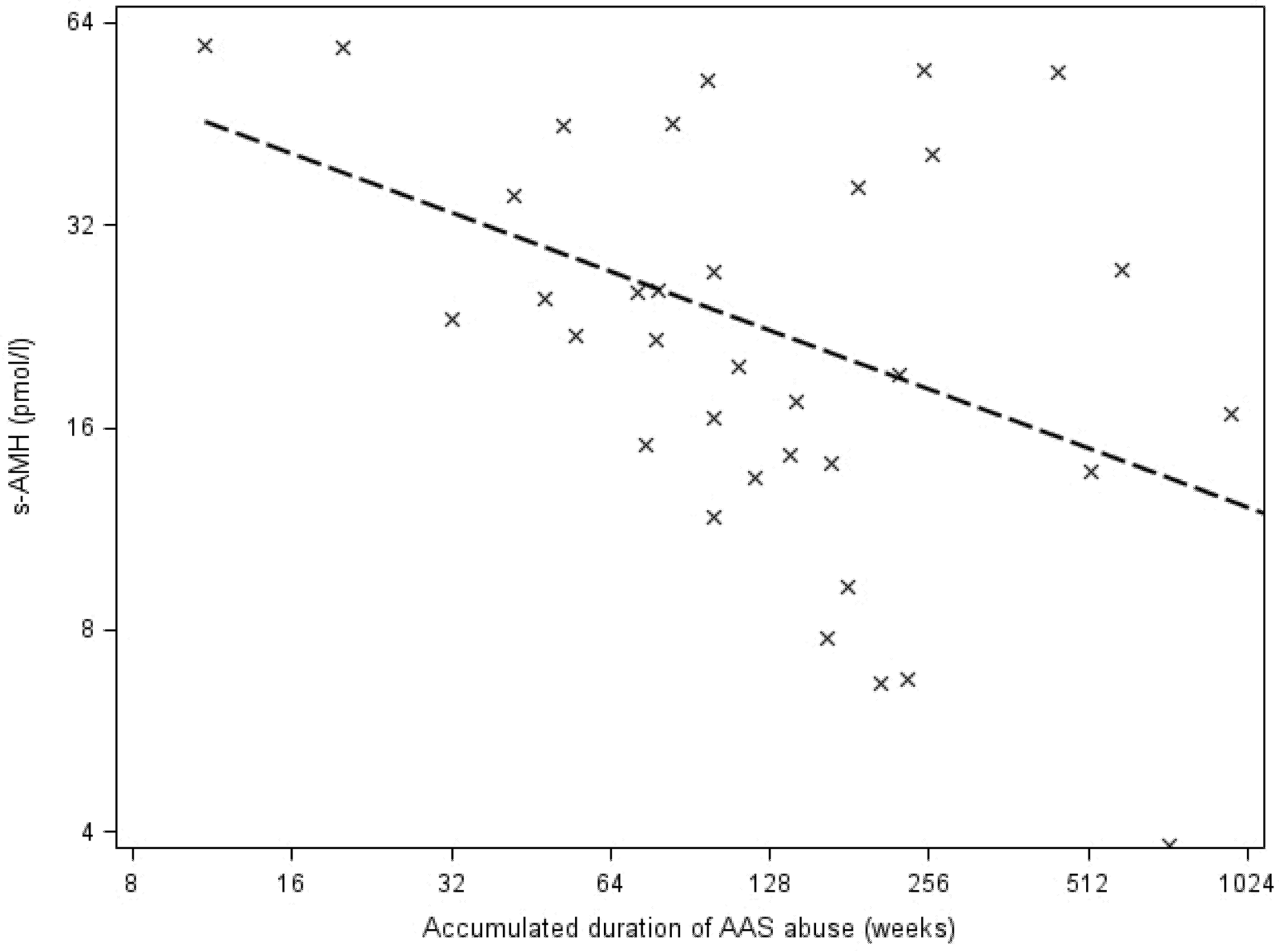
Association between accumulated duration of AAS abuse (log2 scale) and serum anti-Müllerian hormone levels (AMH, log2 scale) in current AAS abusers. **Footnote: AAS,** anabolic androgenic steroids.

Among current AAS abusers, increasing accumulated duration of AAS abuse was associated with decreasing serum inhibin B levels, which reached a plateau after 64 weeks of accumulated AAS abuse (spline function, log2 coefficient (B): -47.9 (-80.3; -15.6), P < 0.01) (Fig 4). Increasing accumulated duration of AAS abuse was also associated with decreasing AMH levels among current AAS abusers (log2 coefficient (B): -0.3 (-0.5; -0.04), P = 0.03) (Fig 5).

### Symptoms Indicating Hypogonadism

**Fig 6:**
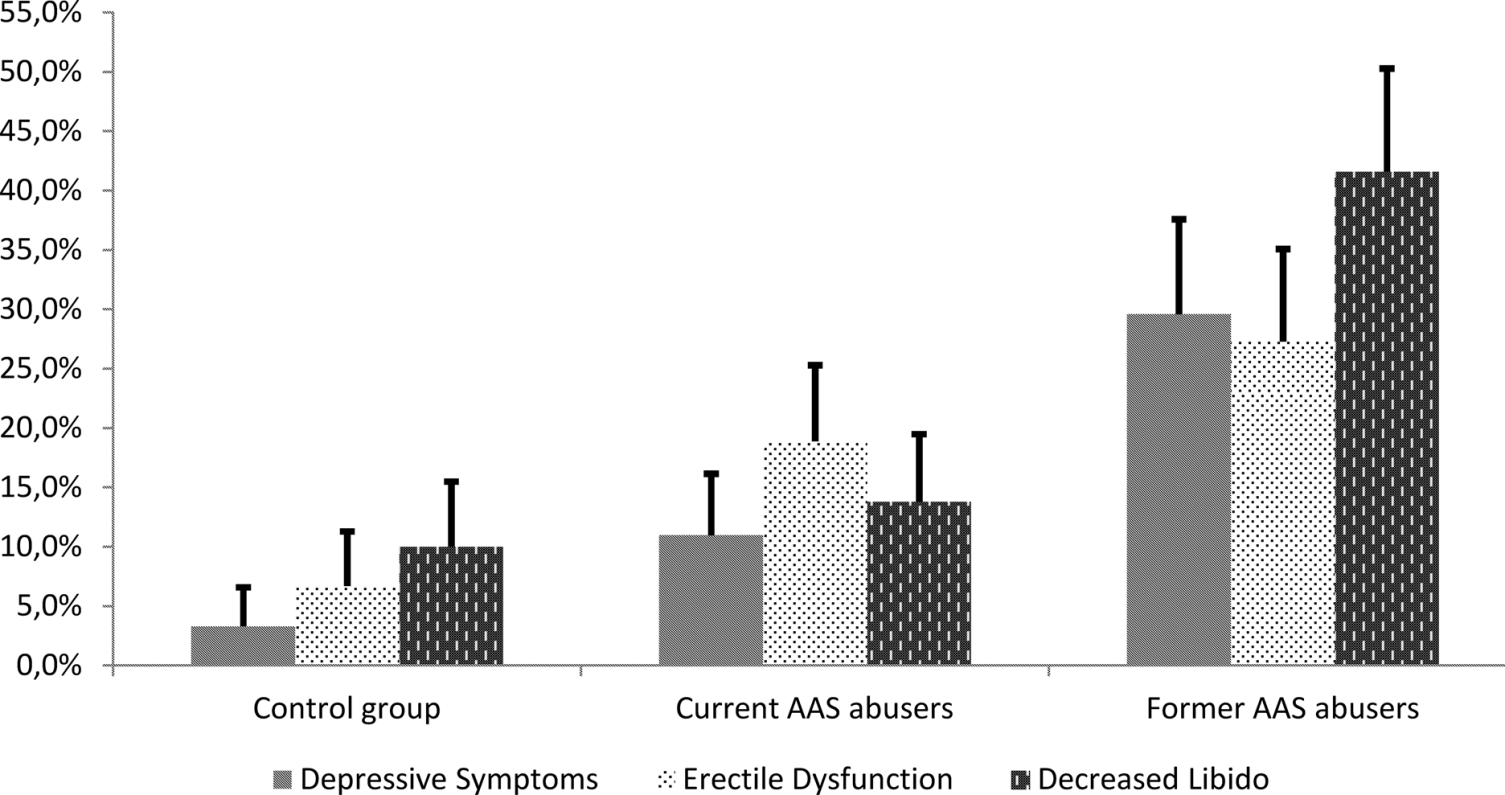
Symptoms of depression, erectile dysfunction and decreased libido in the three groups. **Footnote:** T bars show standard errors. Depressive symptoms, erectile dysfunction and decreased libido were compared across the groups with trend analyses and all were statistically significant (P < 0.05). **AAS,** anabolic androgenic steroids.

Former AAS abusers exhibited the highest frequencies of participants with depressive symptoms (24.2% (11.1; 42.2)), erectile dysfunction (27.3% (13.3; 45.6)) and decreased libido (40.1% (23.2; 57.0)) compared with the other two groups (trend analyses: P < 0.05 for all three parameters) (Fig 6, S2 Table). Former AAS abusers had a lower score on the SF-36 questionnaire with respect to ‘energy/fatigue’ (58.9 (4.3)) than the control group (73.5 (2.6)) and current AAS abusers (69.2 (4.5)) indicating former AAS abusers exhibited significantly more pronounced fatigue symptoms than their counterparts (P < 0.05). We did not observe any significant associations between symptoms and hormonal levels or extent of AAS abuse among former AAS abusers.

## Discussion

The key findings of this study were that the group of former AAS abusers exhibited significantly lower plasma total and free testosterone, smaller testicular sizes, and featured a higher proportion of participants with depressive symptoms, fatigue, erectile dysfunction and decreased libido than the control group more than two years after AAS cessation. These results indicate that a considerable proportion of former AAS abusers exhibited persistent ASIH features, such as biochemical and functional hypogonadism, years after AAS cessation.

We assessed percentages of the groups of control participants and former AAS abusers below the reference limit for plasma total testosterone using reference ranges for both a subgroup of nonobese eugonadal healthy young men (12.1 nmol/l) and a pooled population-representative cohort (6.6 nmol/l). Our findings were that a high proportion of former AAS abusers were below the reference limit for eugonadal nonobese healthy young men compared with none of the control participants, but only 3.3% of former AAS abusers were below the lower reference limit using the pooled population-representative cohort estimation. These findings suggest a rather high proportion of former AAS abusers exhibit testosterone levels in the low area of the normal range years after AAS cessation, whereas only a small proportion of former AAS abusers exhibit persistently marked low testosterone levels.

Serum inhibin B and AMH were markedly decreased among current AAS abusers, but we did not observe differences between former AAS abusers and control participants in these Sertoli-cell biomarkers. Nevertheless, accumulated duration of AAS abuse was strongly associated with decreasing levels of inhibin B and AMH, suggesting the extent of AAS abuse may be important with respect to spermatogenesis recovery and that it may increase the risk of permanent fertility impairment as shown in previously reported cases [10, 12–15]. A higher percentage of former AAS abusers exhibited inhibin B levels suggestive of impaired spermatogenesis than control participants, although the difference was not statistically significant. Nevertheless, this difference may have impacted fertility among former AAS abusers at a population level. Furthermore, it is possible that post-cycle therapy may have reduced the frequency of impaired spermatogenesis in the group of former AAS abusers.

We noted a high proportion of former AAS abusers exhibiting symptoms suggestive of functional hypogonadism. We did not observe any associations between these symptoms and reproductive hormone levels. Previous studies have shown supraphysiologic doses of testosterone (≥ 500 mg/week) occasionally induce hypomania or mania in healthy young men and that rapid decreases in testosterone levels can cause depressive symptoms and decreased libido [32, 33]. The symptoms we observed among former AAS abusers may have been a consequence of abrupt decreases in plasma androgens, from supraphysiologic levels to low or normal levels, following AAS withdrawal, as opposed to specific plasma testosterone levels. Kanayama et al. introduced the term “muscle dysmorphia” and noted it as being highly prevalent among AAS abusers and a cause of dependence [34]. Declines in muscle mass resulting in a more normal body composition, may have caused body image concerns among former AAS abusers in this study as well as functional symptoms of hypogonadism, after AAS cessation. It is also a possibility that former AAS abusers exhibited symptoms consistent with depression and sexual dysfunction before they started using AAS and their symptoms relapsed following AAS cessation.

The results of the present study are generally consistent with those of the recent study by Kanayama et al., who reported even lower plasma testosterone levels than this study, as well as comparable frequencies of hypogonadal symptoms, despite a longer elapsed interval since AAS cessation [16]. A larger proportion of participants (37%) in their study reported that less than 12 months had elapsed since AAS cessation.

To our knowledge, no studies have previously investigated the fertility or biomarkers of Sertoli-cell function in former AAS abusers. Two smaller studies measured serum inhibin B in current AAS abusers and reported levels similar to those measured in current AAS abusers in this study [6, 7]. A few minor studies have investigated sperm counts and morphology in current AAS abusers only and noted severe impairment [35, 36].

This study had several limitations which should be addressed. We interpreted the results in this study using a pseudo-longitudinal approach, but the cross-sectional study design limited our ability to determine causality. Longitudinal participant androgen level monitoring and repetitive urine testing for AAS metabolites would have been ideal, as intermittent AAS abuse is not uncommon among individuals who have stopped using AAS, and we cannot exclude the possibility that the decreased testosterone levels and higher frequencies of hypogonadal symptoms, noted among former AAS abusers in this study, were signs of intermittent AAS abuse and thus indicative of the fact that a much shorter time interval had elapsed since AAS cessation than those reported by the participants. We also have no evidence that participants in the groups of current and former AAS abusers were similar to the control group before starting AAS abuse. Therefore, the results of this study may simply reflect differences among three groups that were already present at baseline. However, the characteristics of the three groups were generally comparable with respect to important demographic parameters. Additionally, participants were recruited from the same communities, which were primarily located in the greater Copenhagen area. The participants volunteered from the community and were not patients from our clinic, but we cannot exclude the possibility that this study may have been affected by selection bias. Recall bias may also have affected our results, as considerable amounts of data were obtained via self-reported histories. We did not screen the urine of the participants for AAS metabolites, but plasma SHBG levels have previously been shown to decrease rapidly during short-term supplementation with the oral AAS, stanozolol, in young healthy men and women [37, 38]. In this study all former AAS abusers exhibited plasma SHBG within the normal reference range and excludes that oral AAS were abused in this group while injections with testosterone could possibly still have been used. We did not obtain sperm samples which could have provided valuable information regarding fertility among the participants. Sexual dysfunction was frequently noted among former AAS abusers and could have biased the semen results, as these participants would likely not have been able to provide semen samples or may have even refused to participate in the study.

In conclusion, the present study showed that a high proportion of former AAS abusers exhibited biochemical and functional ASIH several years after AAS cessation. Current AAS abusers exhibited biochemical abnormalities suggestive of impaired spermatogenesis, which were associated with increasing accumulated duration of AAS abuse. ASIH may become a public health concern with respect to male infertility and hypogonadism.

## Supporting Information

S1 Table Anabolic androgenic steroids (AAS) compounds used by current and former AAS abusers. (DOCX)

S2 Table Symptoms suggestive of functional hypogonadism in the three groups. (DOCX)
