# Supplementary material for: Former Abusers of Anabolic Androgenic Steroids Exhibit Decreased Testosterone Levels and Hypogonadal Symptoms Years after Cessation: A Case-Control Study
Source: PLoS One. 2016 Aug 17;11(8):e0161208. doi: 10.1371/journal.pone.0161208 (PMC4988681; doi:10.1371/journal.pone.0161208)
Supplement: S1 Table — (DOCX) [file pone.0161208.s001.docx]

|  |  |  |  |  |  |
| --- | --- | --- | --- | --- | --- |
|  | **Current AAS abusers** | | **Former AAS abusers** | | **p-value** |
| **Compounds** |  | dose range per week (mg) |  | dose range per week (mg) |  |
| Testosterone esters (%) | 97.3 | 500 - 2000 | 96.3 | 500 - 1500 | 1.00 |
| Trenbolone (%) | 78.4 | 250 - 750 | 57.6 | 250 - 1000 | 0.08 |
| Nandrolone (%) | 73.0 | 250 - 1000 | 78.8 | 250 - 1000 | 0.48 |
| Stanozolol (%) | 73.0 | 200 - 700 | 66.7 | 200 - 700 | 0.57 |
| Sustanon (%) | 70.2 | 750 - 2000 | 54.5 | 500 - 1000 | 0.20 |
| Boldenone (%) | 64.9 | 600 - 2000 | 27.3 | 500 - 750 | <0.01 |
| Drostanolone (%) | 62.2 | 300 - 700 | 45.5 | 300 - 500 | 0.23 |
| Metenolone (%) | 59.5 | 400 - 1000 | 54.6 | 400 - 1000 | 0.72 |
| Oxandrolone (%) | 56.8 | 350 - 700 | 42.4 | 350 - 420 | 0.28 |
| Methanolone (%) | 48.7 | 280 - 350 | 51.5 | 350 - 700 | 0.72 |
| Oxymetholome (%) | 27.0 | 350 - 700 | 57.6 | 350 - 700 | <0.01 |
| Clostebol (%) | 10.8 | 160 - 280 | 6.1 | not reported | 0.83 |
| Gestrinone (%) | 5.4 | not reported | 9.1 | not reported | 0.15 |
| Metribolone (%) | 2.7 | not reported | 6.1 | 200 - 600 | 0.41 |
| Androstenione (%) | 0.0 | - | 3.0 | not reported | 0.73 |
|  |  |  |  |  |  |
|  |  |  |  |  |  |

**AAS,** anabolic androgenic steroids

p-values are generated as response to chi-square test or Fisher’s exact test as appropiate.
