# Supplementary material for: Former Abusers of Anabolic Androgenic Steroids Exhibit Decreased Testosterone Levels and Hypogonadal Symptoms Years after Cessation: A Case-Control Study
Source: PLoS One. 2016 Aug 17;11(8):e0161208. doi: 10.1371/journal.pone.0161208 (PMC4988681; doi:10.1371/journal.pone.0161208)
Supplement: S2 Table — (DOCX) [file pone.0161208.s002.docx]

|  |  | **BDI II (depressive symptoms)** |  |  |
| --- | --- | --- | --- | --- |
|  | **Control group** | **Current AAS abusers** | **Former AAS abusers** | **p-value** |
| normal (n, (%)) | 30 (96.7) | 32 (88.9) | 25 (75.0) | 0.03 |
| mild mood disturbance (n, (%)) | 1 (3.3) | 3 (8.3) | 2 (6.3) |  |
| Borderline depressive symptoms (n, (%)) | 0 | 0 | 4 (12.5) |  |
| moderate depressive symptoms (n, (%)) | 0 | 1 (2.8) | 0 |  |
| severe depressive symptoms (n, (%)) | 0 | 0 | 2 (6.3) |  |
|  |  |  |  |  |
|  |  | **Libido (question 21)** |  |  |
|  | **Control group** | **Current AAS abusers** | **Former AAS abusers** | **p-value** |
| not any change (n, (%)) | 28 (90.3) | 31 (86.1) | 19 (59.4) | 0.02 |
| less interested in sex than I used to be (n, (%)) | 3 (9.7) | 3 (8.3) | 10 (31.3) |  |
| much less interestedin sex now (n, (%)) | 0 | 2 (5.6) | 2 (6.3) |  |
| I have completed lost interest in sex (n, (%)) | 0 | 0 | 1 (3.1) |  |
|  |  |  |  |  |
|  |  | **IIEF-5 (erectile dysfunction)** |  |  |
|  | **Control group** | **Current AAS abusers** | **Former AAS abusers** | **p-value** |
| Normal (≥ 22 points) (n, (%)) | 29 (93.6) | 30 (81.1) | 24 (72.3) | 0.03 |
| Mild dysfunction (17 - 21 points) (n, (%)) | 2 (6.7) | 4 (10.8) | 7 (21.1) |  |
| Moderate dysfunction (8 - 16 points) (n, (%)) | 0 | 3 (8.1) | 2 (6.1) |  |
| severe dysfunction (≤ 7 points) (n, (%)) | 0 | 0 | 0 |  |
|  |  |  |  |  |
|  |  | **Erection hardness score (erectile dysfunction)** |  |  |
|  | **Control group** | **Current AAS users** | **Former AAS users** | **p-value** |
| **4 (n, (%))** | 30 (96.8) | 27 (73.0) | 22 (66.7) | 0.02 |
| **3 (n, (%))** | 1 (3.2) | 10 (27.0) | 6 (18.2) |  |
| **2 (n, (%))** | 0 | 0 | 2 (6.1) |  |
| **1 (n, (%))** | 0 | 0 | 1 (3.0) |  |
| **0 (n, (%))** | 0 | 0 | 2 (6.1) |  |
